# Optimized Strategy for the Control and Prevention of Newly Emerging Influenza Revealed by the Spread Dynamics Model

**DOI:** 10.1371/journal.pone.0084694

**Published:** 2014-01-02

**Authors:** Wen-Dou Zhang, Zheng-Hu Zu, Qing Xu, Zhi-Jing Xu, Jin-Jie Liu, Tao Zheng

**Affiliations:** Center for Biosecurity Strategy Management, Beijing Institute of Biotechnology, Beijing, China; University of Illinois at Urbana-Champaign, United States of America

## Abstract

No matching vaccine is immediately available when a novel influenza strain breaks out. Several nonvaccine-related strategies must be employed to control an influenza epidemic, including antiviral treatment, patient isolation, and immigration detection. This paper presents the development and application of two regional dynamic models of influenza with Pontryagin’s Maximum Principle to determine the optimal control strategies for an epidemic and the corresponding minimum antiviral stockpiles. Antiviral treatment was found to be the most effective measure to control new influenza outbreaks. In the case of inadequate antiviral resources, the preferred approach was the centralized use of antiviral resources in the early stage of the epidemic. Immigration detection was the least cost-effective; however, when used in combination with the other measures, it may play a larger role. The reasonable mix of the three control measures could reduce the number of clinical cases substantially, to achieve the optimal control of new influenza.

## Introduction

2In 2009, influenza A virus subtype H1N1 swept the globe [1]. The highly pathogenic avian influenza subtype H5N1 has threatened humans for many years [2], [3]. Currently, human infection with the avain influenza subtype H7N9 is a serious concern in China, and the risk of new influenza outbreaks is increasing. As the most effective intervention to extinguish influenza, vaccine distribution is among the main directions of research into influenza prevention and control [4], [5]. However, a matching vaccine is not available in the short-term when a new influenza strain breaks out. For example, H1N1 was detected in April 2009 in China, but no matching vaccine was available until September 2009. The epidemic trend of a new influenza strain gradually spreads from one region to all over the world, and in most regions, new influenza epidemics are caused by imported cases.

Given the long vaccine development cycle (up to several months) and the huge gap between vaccine production and demand [6], considerable time is needed for the proportion of immune individuals to be sufficiently large to inhibit the spread of an influenza epidemic. It has become increasingly important to understand how to prevent and control influenza without vaccines [7]–[9]. To this end, three main measures have been employed. Antiviral treatment is an effective approach for treating patients in the early stages of infection [10] and shortening the infectious period. Isolation of infectious patients at specialized hospitals for infectious diseases helps to prevent the spread of an epidemic [11], [12]. Immigration detection involves the detection of influenza (*e.g.*, by measuring body temperature) at ports of entry.

However, these control measures have limitations. For example, poor countries do not sufficient capacity or financial support to produce, stockpile, or update adequate antiviral resources [7]. To be effective, the isolation of infected patients requires the government to provide manpower, materials, and free medical care [13]. Immigration detection can be an expensive endeavor that may affect international trade. Policymakers and government officials need to understand how to optimize control measures and improve their cost-effectiveness for new influenza strains. An inability to develop cost-effective control strategies will result in wasted resources and poor control.

Optimal control theory, which involves determining the optimal solution from among all possible control schemes, has been successfully applied in many areas, such as mechanical control, biology, economics, and so on. Recently, there have been many applications of optimal control theory to the prevention and control of infectious diseases [14]–[17]. Computer simulations based on dynamic models of infectious diseases and optimal control theory provide fast, inexpensive, and effective methods to explore optimal control strategies.

Few studies have addressed how to use the three epidemic control schemes of antiviral treatment, patient isolation, and immigration detection to prevent and control new influenza outbreaks. However, the identification of optimal control strategies to minimize the impact of influenza pandemics and the cost of pandemic control is greatly needed. The aim of the study described in this paper was to develop and apply two regional dynamic models of influenza, to determine the optimal control strategies with the three control measures.

## Models and Methods

### Mathematic Model of Influenza

**Figure 1:**
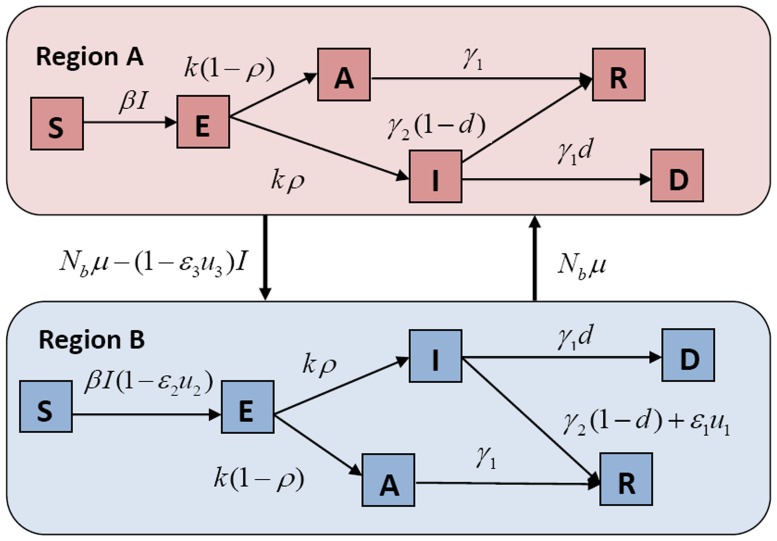
Prevention and control model of new influenza between epidemic region A and nonepidemic region B.

We constructed a dynamic model of interregional influenza transmission that contained the epidemic prevention and control variables, combined with the characters of the classic compartment model of infectious diseases and new influenza protection [18], [19]. To simplify the model, we assumed the existence of two regions: epidemic region A and nonepidemic region B. In the case of no immigrant detection, the proportion of flowing population to the total population 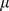 between the two regions was assumed to be constant and equal. We considered the application of the three optimal dynamic control strategies to region B, for the case in which region A does not take any control strategy (see Fig. 1).

In the dynamic model, individuals were classified as susceptible (S), exposed (E), subclinical (A), clinical (I), recovered (R), or dead (D). The dynamic model was given by the following system of nonlinear differential equations:
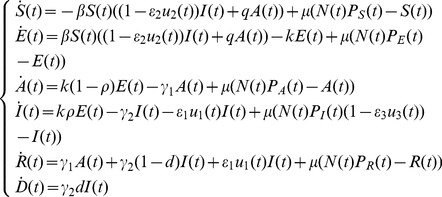
(1)with:


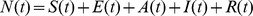
: total population size of region B,


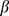
: per-capita transmission rate,


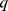
: infectivity ratio between subclinical and clinical patients,


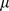
: daily flow of the proportion of the population,


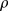
: clinical infection rate,

*d* : mortality rate of clinical patients (all subclinical patients were assumed to recover),


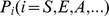
: proportion of the various classes in region A.

*k*: rate of transfer of individuals in class E into infectious class A or I, and


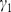
,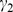: recovery or death rates of individuals in class A and I, respectively.

Because influenza is typically a seasonal disease, and the epidemic period is not very long, we did not consider the natural birth and death rates in our transmission model. 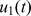, 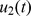, and 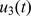 were the intensities of antiviral treatment, patient isolation, and immigration detection, respectively. Because it was difficult to detect an individual in class E or A, we assumed that epidemic intervention could only be used for clinical patients. 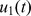 represented the proportion of clinical patients who accepted antiviral treatment (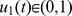), with 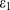 as the efficacy of this treatment (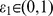). 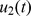 represented the proportion of isolated clinical patients, with 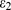 as the efficacy of this isolation (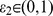). 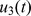
represented the proportion of flowing clinical patients who underwent immigration detection (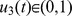), with 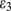
as the efficacy of immigration detection (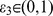). The basic reproduction number *R*_0_ was used to describe the spreading capacity of infectious disease in the disease transmission dynamic model [20]. With no control strategy, the *R*_0_ of model Eq. (1) was given by:
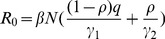
(2)


### Optimal Control

It is difficult to identify and control individuals in classes E and A during an influenza epidemic, and individuals in these classes will not cause direct pressures on the healthcare resources and economic activities. Given these assumptions, we defined the objective function for the cost-effectiveness of the control strategies as:
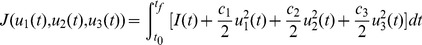
(3)with:


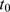
, 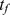: start and end times, respectively, of epidemic prevention and control, and


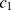
, 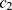, and 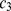: constants to balance the relative costs of interventions and clinical patients.

The goal of the optimal control strategy calculation problem was to find optimal functions 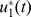, 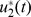, and 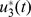, such that the total cost of interventions and clinical patients was lowest, namely:
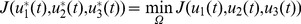
(4)



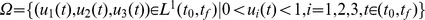
, and 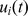 was assumed to be bounded by the Lebesgue integrable function on [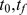] [21]. The problem was translated to optimize the control process of coupled nonlinear differential equations. Pontryagin’s Maximum Principle can be used to solve this problem [22]. The calculation method and process are shown in supporting information: Text S1.

## Results and Discussion

### Scenario

Pandemic data from the 2009 H1N1 outbreak were used to calibrate the parameters in our dynamic model of infectious disease. We made the following assumptions:

1. The total population of region B was 100,000. All of them were susceptible.
2. The influenza epidemic began in region A, and then spread to region B.
3. The upper limits of the intensities of antiviral treatment, immigration detection, and isolation were 0.9, 0.9, and 0.6, respectively. The limit for isolation was lower than the other limits because it was hard to execute effectively. Thus, 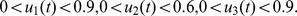

4. According to medical research, both clinical and subclinical infections are possible from the same influenza virus source [23]. There is no research to indicate the specific proportion of clinical infection of influenza A (H1N1). We speculate that the number of clinical cases is approximately equal to the number of subclinical cases from literature [24], [25]. Therefore, we assumed that 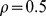.
5. The infectivity and infectious period of subclinical individuals have not been statistically validated with medical observations. Because these patients lack coughing, sneezing, and other obvious influenza symptoms, their infectivity will be much weaker than that of clinical individuals. We assumed that the infectious period for a subclinical individual was 5 days, and we used infectious parameters from the 1918 influenza outbreak [26].
6. Three weight values were defined to balance the cost of control measures and loss of influenza: 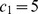,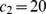, and 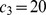.

Table 1 displays the specific values, meanings, and sources of the parameters used in the study. The population mobility and the weights and effectiveness levels of the different control measures were difficult to determine and were subjected to sensitivity analyses. The simulation time was set to 200 days. The infectivity levels of various new influenza strains may differ considerably. For example, the basic reproduction number *R*_0_ was about 3.75 for the Spanish flu in the autumn 1918 epidemic period, but *R*_0_ was 1.5 for the 2009 H1N1 epidemic [25], [29]. We assumed an *R*_0_ for new influenza of 3.0. The transmission rate 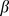 can be solved from Eq. (2) (Other assumptions see supporting information: Text S3).

**Table 1:** Model parameter definitions and values in simulation model.

Parameter	Description	Value	References
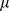	Mobility of the population between regions A and B	0.003	Assumption
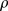	Clinical infection rate	0.5	[24], [25]
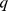	Relative infectivity of the subclinical individual	0.003	[26]
1/k	Mean incubation period (days)	2	[27]
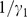	Mean infectious period of subclinical infection (d)	5	Assumption
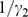	Mean infectious period of clinical infection (d)	6	[27], [28]
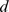	Mortality rate of clinical infection	0.004	[25]
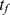	Simulation duration (d)	200	Assumption
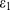	Efficacy of antiviral treatment	1/6	[10]
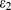	Efficacy of isolation	0.5	Assumption
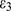	Efficacy of immigration detection	0.5	Assumption

### Numerical Results

**Figure 2:**
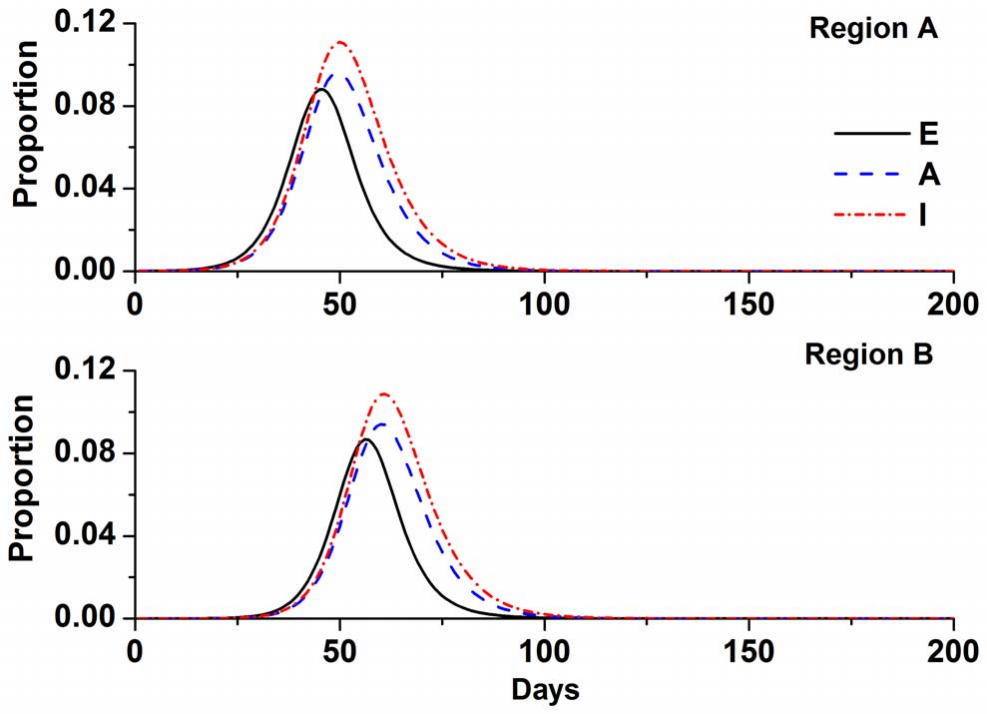
Epidemic trends without a control strategy in region B. Simulation parameters are shown in Table 1. 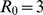.

Fig. 2 shows the epidemic trends analyzed without the use of a control strategy in region B. Under this condition, the epidemic trends and infection rates of regions B and A were similar, except with a delay of 12 days. The proportion of all people who were clinically infected over the total 200 days was 45.41%. The peak proportion of clinically infected individuals was 11.09%.

**Figure 3:**
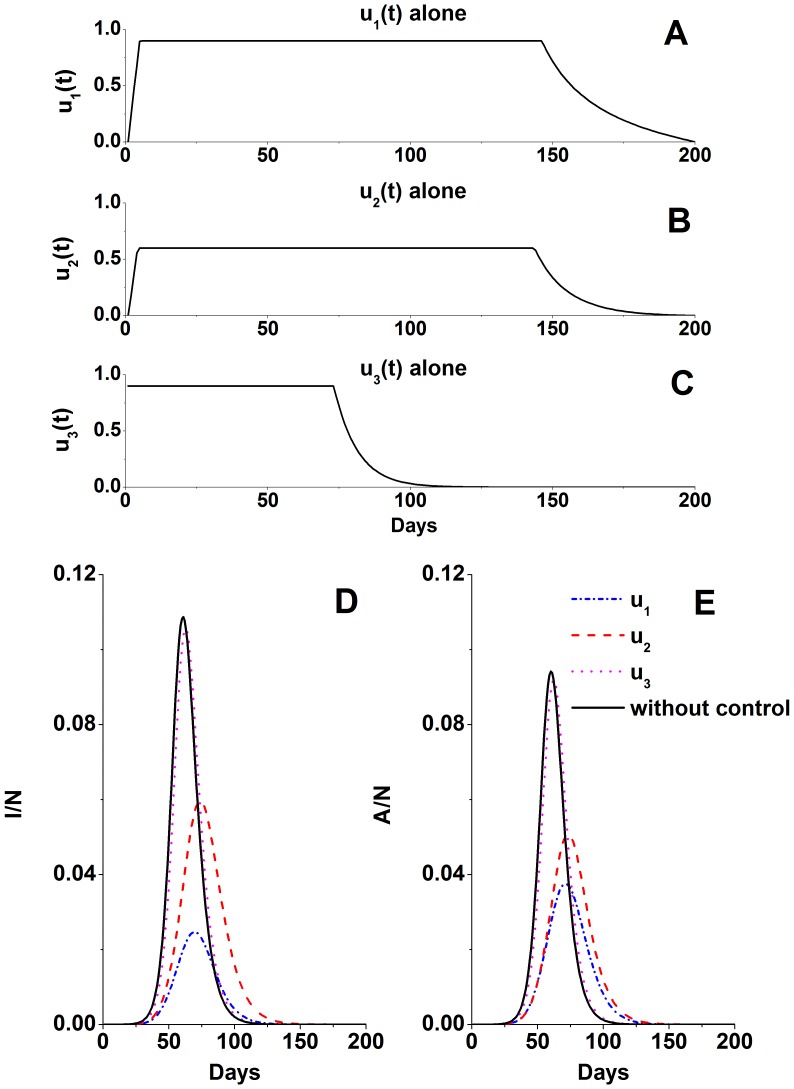
Optimal control and epidemic trend functions of region B, using antiviral treatment, patient isolation, or immigration detection alone. **A–C**: Optimal control functions. **D**, **E**: Functions of the clinical and subclinical patients. Simulation parameters are shown in Table 1. 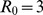.

Next, we explored the optimal control strategy functions of region B using only one control measure: antiviral treatment, patient isolation, or immigration detection (Fig. 3). Antiviral treatment was the most effective measure to control clinical infection. Its use reduced the total and peak proportions of clinically infected individuals to 15.07% and 2.47%, respectively. According the Eq. (10) (supporting information: Text S1), the antiviral resource demand is one day of doses for 812 persons (812 DDs) per thousand people for 200 days. Patient isolation reduced the total and peak proportions of clinically infected individuals to 37.13% and 5.97%, respectively. Isolation prolonged the duration of an epidemic; therefore, although it was useful to control the peak of an epidemic, it did not control the total number of clinical patients. The total and peak proportions of clinically infected patients were 44.68% and 10.87%, respectively, when immigration detection was used.

**Figure 4:**
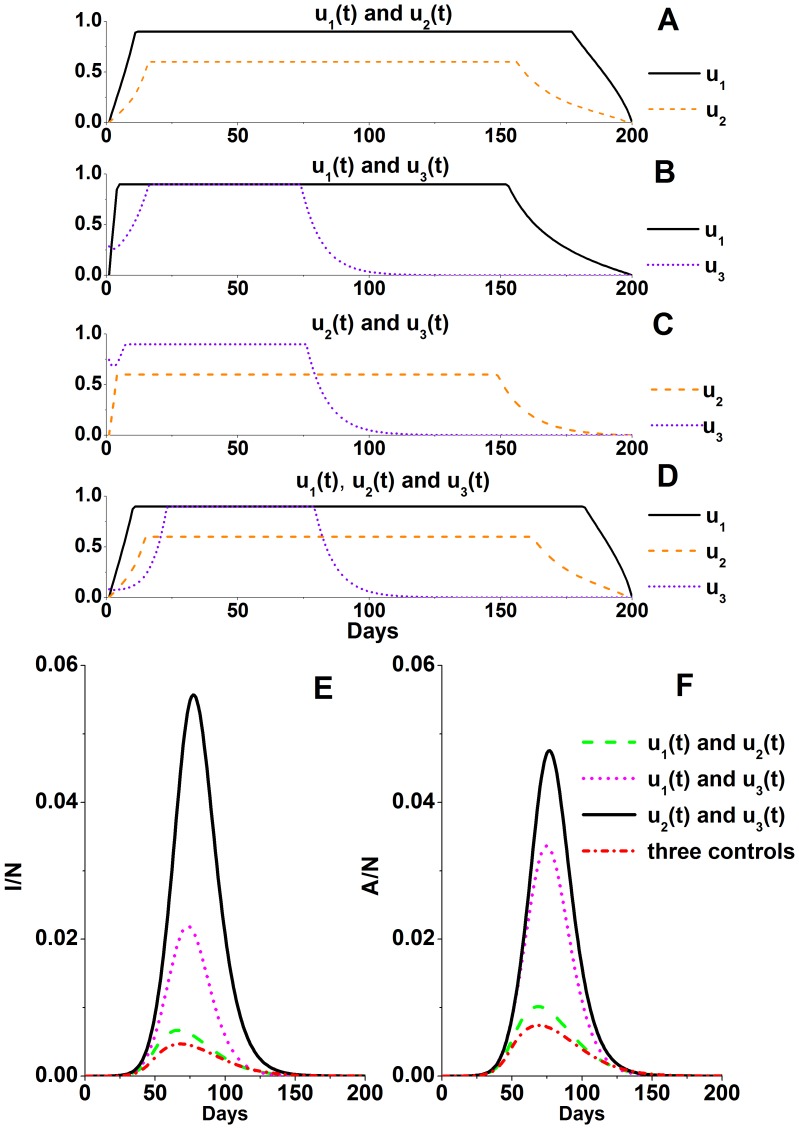
Optimal control and epidemic trend functions of region B, using mixed epidemic control strategies. **A–D**: Optimal control functions. **E, F**: Functions of the clinical and subclinical patients. Simulation parameters are shown in Table 1. 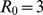.

Finally, we explored the optimal control strategy functions of region B using mixed control strategies with two or three control measures (Fig. 4). A comparison of Fig. 3 and 4 clearly shows that the use of a mixture of control strategies was more effective than the use of a single strategy for controlling an epidemic. The use of mixed control strategies reduced the total and peak proportions of clinically infected individuals to 4.46% and 0.47%, respectively. The antiviral drug demand was 241 DDs per thousand people. The more effective the control strategy was, the longer it was used at high intensity, to avoid a second outbreak of the epidemic.

### Sensitivity Analysis

**Figure 5:**
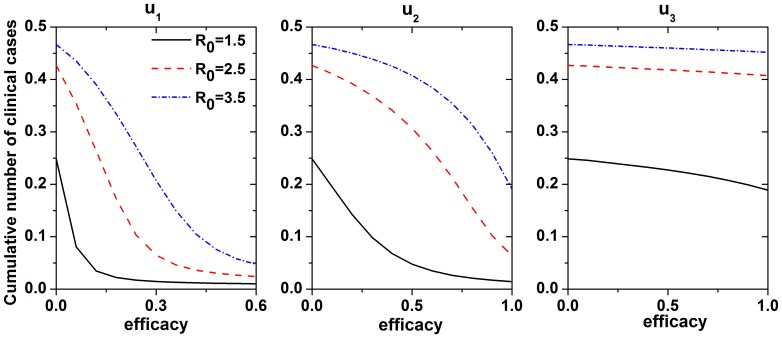
Cumulative number of clinical cases (vertical axis) as a function of the efficacy of individual prevention measures (horizontal axis) with different 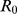 values. Simulation parameters except 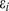 are shown in Table 1.

We investigated the role of the control measure parameters with different *R*_0_ values via sensitivity analysis. Fig. 5 shows the cumulative number of clinical cases as a function of the efficacy of each control measure with different values of *R*_0_. The efficacy of antiviral treatment was the most sensitive of the control measures to the cumulative number of clinical cases. When the efficacy was 0.5 (*i.e*., antiviral treatment shortened the infectious period of symptoms to 2 days), this control measure could effectively control influenza, especially virus with low infectivity. The efficacy of patient isolation was positively correlated with *R*_0_, such that stricter control was needed as the infectivity of the influenza increased.

**Figure 6:**
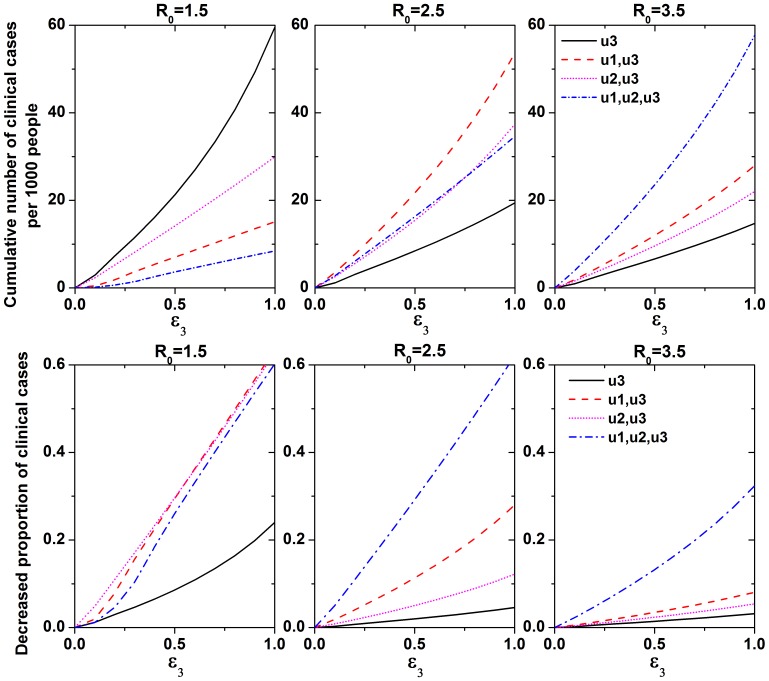
Effectiveness analysis of immigration detection. **Top:** Cumulative reduction in the number of clinical cases per thousand persons. **Bottom:** Reduction in the proportion of clinical cases as a function of the efficacy of control measures under different 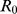 values.

Little research is available on the use of immigration detection as a control measure for influenza epidemics. Because there were many exposed and subclinical cases, this measure only reduced the number of cumulative infected cases by a small amount. However, immigration detection also depended on the mobility of the population between the two regions. To explore the effectiveness of immigration detection further, we compared the cumulative number of clinical cases when immigration detection was used alone or with other control measures (Fig. 6). Immigration detection was more effective when used in conjunction with other control measures.

**Figure 7:**
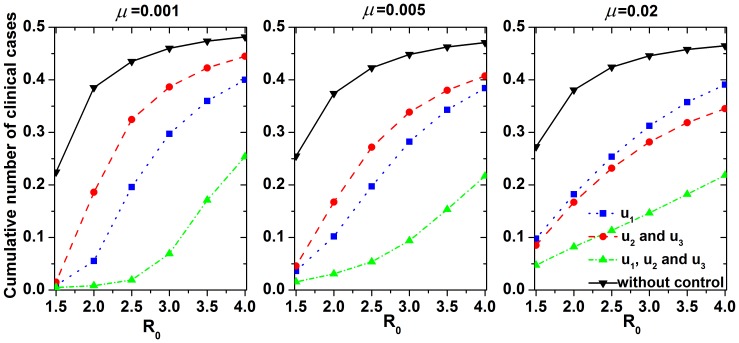
Impact of population mobility on epidemic control. Cumulative number of clinical cases in region B is shown as a function of 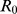 for strategies 1, 2, and 3 when the population mobility is 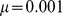, 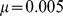, and 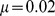, respectively.

Based on the above findings, to simplify our calculations, we focused on the following three control strategies: antiviral treatment *u*_1_ alone (strategy 1), nonpharmacologic interventions *u*_2_ and *u*_3_ (strategy 2), and pharmacologic and nonpharmacologic interventions *u*_1_, *u*_2_, and *u*_3_ (strategy 3). The mobility of population 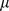 directly resulted in the spread of disease from the epidemic to the nonepidemic region. Therefore, we analyzed the impact of population mobility on epidemic control. With the increased population mobility, immigration detection increased in importance, and nonpharmacologic intervention was more effective than antiviral treatment (Fig. 7).

**Figure 8:**
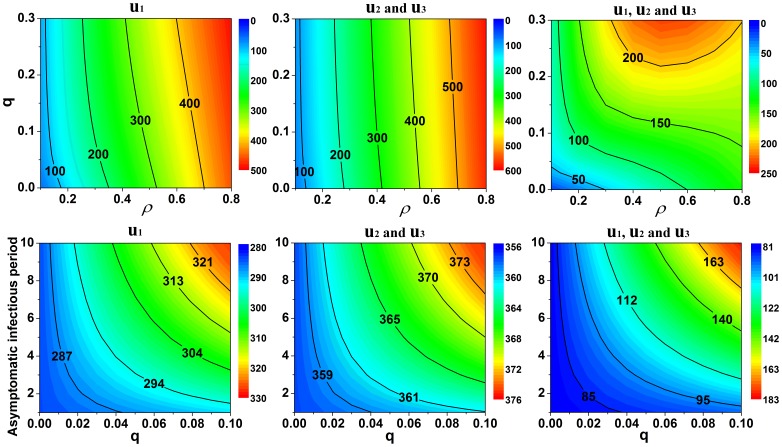
Epidemic trend effects, in terms of the infectious period of clinical cases, relative Infectivity, and proportion of clinical infection. Cumulative number of clinical cases per thousand people as a function of *q* and 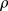 (top) or 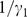 and *q* (bottom).

Subclinical cases are difficult to track, and their role in the transmission dynamics of infectious diseases is difficult to confirm. Hsu *et al.* explored these issues by mathematical derivation [30]. We performed sensitivity analyses of three key parameters of the subclinical cases: the infectious period 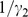, relative infectivity *q*, and proportion of clinical infectious individuals 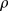 (Fig. 8). Compared to the other parameters, the effects of 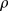 on the cumulative number of clinical cases was the most significant. We observed a negative correlation between the effects of 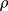 and the intensities of the control strategies. As *q* increased, the effects of 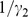 on the cumulative number of clinical cases also increased.

Due to fear of infection, the migration rates may spontaneous decrease during an epidemic period. To test whether this change influences the simulation results, we performed a sensitivity analysis. We examined decreasing migration rates in the range of 0 to 0.8 with different values of *R*_0_ (see supporting information: Text S4, Fig. S1, Fig. S2, Fig. S3). The cumulative number of clinical cases was not strongly affected by migration rates. However, when the control strategies were relatively effective (i.e., cumulative number of clinical cases per thousand people <100), the proportional change in the number of clinical cases was significant, and the number of clinical cases was positively correlated with the migration rate. When the control strategies were not effective (i.e., cumulative number of clinical cases per thousand people >100), the result was the opposite.

**Figure 9:**
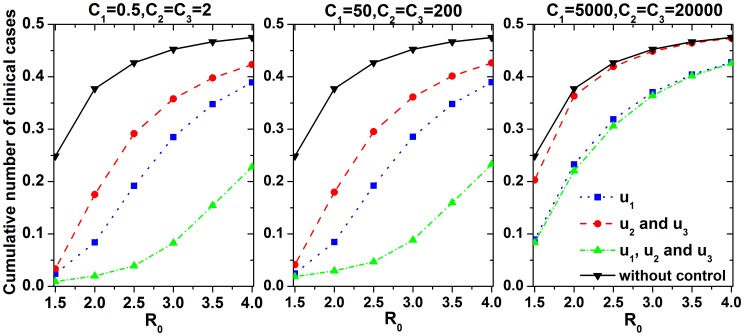
Comparison of cumulative clinical cases for optimal control strategies with different 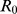 and weight values 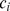.

The costs of clinical cases and control strategies are impacted by the influenza epidemic level and regional economic conditions. Fig. 9 shows the effects of parameters 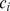, which were used to balance these costs. Epidemic control was not obviously affected by 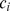 when the cost-effectiveness of the control measures was good (*i.e.*, 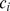 were not sufficiently large). However, once 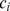 increased to a certain value (cost of control strategies>loss of clinical cases), the optimal strategies tended to reduce the intensities of control measures, especially less cost-effective nonpharmacologic measures, and the cumulative number of clinical cases increased substantially. Therefore, reducing the cost of control measures can ensure a good control when the optimization goal is to maximize the cost-effectiveness.

**Figure 10:**
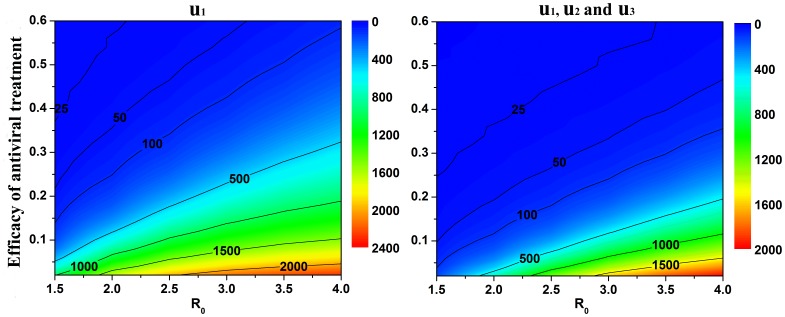
Relationship between the efficacy of antiviral treatment and the optimal antiviral stockpile (per thousand people), with different 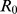 values. Colors represent the optimal antiviral stockpile (DDs per thousand people) under different efficacies of antiviral treatment.

The emergency production capacity of antiviral resources is inadequate in many countries and regions. Therefore, how many antiviral resources are stockpiled for a new influenza outbreak is a wide concern. We explored the role of antiviral resources in the optimal control strategies (Fig. 10). The optimal antiviral stockpile was very sensitive to the efficacy of the antiviral treatment. Thus, the use of effective and generally applicable antiviral drugs was important for controlling the extent and cost of new influenza epidemics.

### Optimal Control Strategies with Limited Antiviral Resources

**Figure 11:**
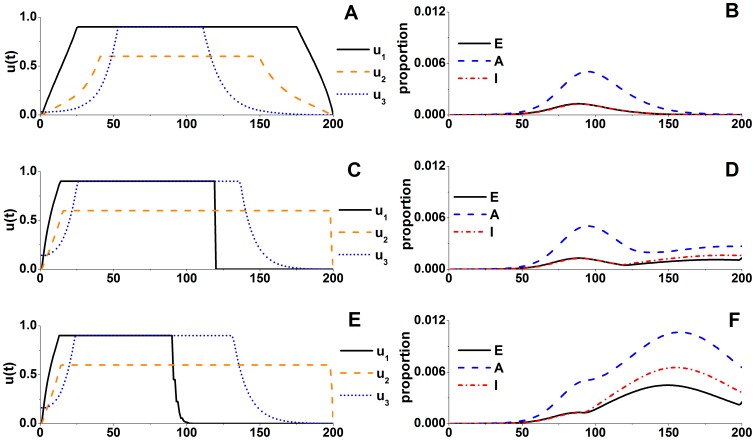
Optimal control strategies and epidemic trends in the case of limited antiviral resources. **A**, **B**: Curves of control strategies and epidemic trends, respectively, in the case of adequate antiviral resources. **C–F**: Curves of control strategies (C, E) and epidemic trends (D, F) when antiviral resources are 50 DDs (C, D) and 30 DDs (E, F) per thousand people. 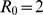.

We modified the mathematical and optimal control models to explore the optimal control strategies in the scenario of limited antiviral resources (see supporting information: Text S2).Without loss of generality, we restrict our discussion to the situation with 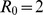. Under conditions of adequate resources, antiviral stockpiles to achieve optimal control strategies should satisfy 60 DDs per thousand people. Fig. 11 shows the results for the optimal control strategies under scenarios with insufficient antiviral stockpiles: namely, for 50 and 30 DDs per thousand people. Maximum-intensity implementation of antiviral treatment in the early stage of the pandemic until the antiviral stockpiles are exhausted was observed to be the optimal control strategy under conditions of inadequate antiviral resources. Intense nonpharmacologic interventions should also be used. However, when antiviral resources are exhausted, an epidemic is hard to control by nonpharmacologic interventions, and a second outbreak will occur.

## Conclusion

In this paper, we explored the optimal control strategies for nonepidemic regions to prevent and control the spread of new influenza strains in the absence of matching vaccines. We found that the prompt use of adequate antiviral stockpiles can effectively reduce the peak and cumulative numbers of clinical cases by shortening the infectious period of influenza. Antiviral treatment was generally a more cost-effective control measure than nonpharmacologic measures, making it one of the most cost-effective measures to prevent influenza except vaccines. Improving the efficacy of antiviral drugs was a key method to reducing the size of antiviral stockpiles and the cost of antiviral treatment.

The isolation of clinical cases was useful for controlling the peak of the epidemic. Use of this measure combined with antiviral treatment could be suitable, especially when antiviral stockpiles are inadequate. Immigration detection cannot detect all clinical cases [31], and is particularly poor at detecting exposed and subclinical cases. Immigration detection could not prevent the spread of an epidemic. It could only reduce the number of imported cases to a limited extent, and it was the least cost-effective measure. It was more effective when used in conjunction with other control measures, compared to when used alone. The use of a mixed strategy with all three control measures was the most effective approach, reducing the cumulative and peak proportions of clinical cases by more than 90% and 95%, respectively, when 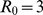. A spontaneous decrease in migration rates can reduce the proportion of clinical cases, when strict and effective control strategies are in place.

In many regions, inadequate antiviral resources are available to meet the demands of an influenza epidemic. In this case, we found that the optimal strategy was to concentrate the use of antiviral resources during the early stage of the epidemic, and to improve the intensity of the nonpharmacologic controls. For example, antiviral resources should be reasonably distributed to hospitals as soon as possible after a new influenza outbreak, to maximize the intensity of antiviral treatment in the early stage of the pandemic until the antiviral stockpiles run out.

We used a mathematic model to explore the optimal control strategies of new influenza in a nonepidemic region, but the situation is more complicated in the actual decision-making process. For example, not all individuals were equally susceptible to the 2009 influenza A subtype H1N1. Studies have shown that about 10% of the population has a natural immunity to H1N1, and the proportion of immunity in older people is as high as 33% [24], [32]. High proportions of infected children, young people, and pregnant women were observed in the 2009 outbreak, and 60% of patients were 18 years of age or younger [33]. Moreover, antiviral drugs can only be effectively used during a limited window in the disease course. For the 2009 H1N1 strain, to be effective, an antiviral drug had to be used within the first 48 hours after infection [34].

Although we were unable to build a precise model of epidemic control to describe all of these details, these limitations do not preclude us from using optimal control theory to study the basic control of new influenza outbreaks by building a rational model and adjusting the parameters. Based on the results from these models, we can, to some extent, predict the epidemic trends and make optimal plans for the control of new influenza strains. We believe that these simulation results may have important significance for departments of epidemic control: for example, in guiding the determination of antiviral stockpiles and optimal control strategies.

## Supporting Information

Figure S1
**Cumulative number of and change in the proportion of clinical cases in region B as a function of migration rates between regions A and B, when** 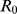 **= 1.5.** Simulation parameters except 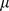 are shown in Table 1.(TIF)

Figure S2
**Cumulative number of and change in the proportion of clinical cases in region B as a function of migration rates between regions A and B, when** 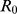 **= 2.5.** Simulation parameters except 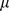 are shown in Table 1.(TIF)

Figure S3
**Cumulative number of and change in the proportion of clinical cases in region B as a function of migration rates between regions A and B, when** 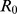 **= 3.5.** Simulation parameters except 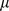 are shown in Table 1.(TIF)

Text S1 **The calculation method of Pontryagin’s Maximum Principle with unlimited antiviral resources.**
(PDF)

Text S2 **The calculation method of Pontryagin’s Maximum Principle with limited antiviral resources.**
(PDF)

Text S3 **Basic assumptions of model.**
(PDF)

Text S4 **Sensitivity analysis of migration rates.**
(DOC)
