# Correction: Optimized Strategy for the Control and Prevention of Newly Emerging Influenza Revealed by the Spread Dynamics Model

**DOI:** 10.1371/annotation/8254081b-b193-4799-9ddf-d9a71d2007b4

**Published:** 2014-01-22

**Authors:** Wen-Dou Zhang, Zheng-Hu Zu, Qing Xu, Zhi-Jing Xu, Jin-Jie Liu, Tao Zheng

The first two authors, Wen-Dou Zhang and Zheng-Hu Zu, should be noted as joint first authors of this work.

There were errors in the Funding section. The correct funding statement is as follows: 

"Funding was provided by Mega-Project on Infectious Disease Prevention of China (No. 2013ZX10004-605, No. 2012ZX10004-402). National High Technology Research and Development Program of China(863 Program) (No. 2012AA022007). Medical Science and Technology Research Projects of China (No. AWS11L009, No. 13QNP156). National Natural Science Foundation of China (NSFC) (No. 90924019). The funders had no role in study design, data collection and analysis, decision to publish, or preparation of the manuscript.

