# Supplementary figures and images for: Optimized Strategy for the Control and Prevention of Newly Emerging Influenza Revealed by the Spread Dynamics Model
Source: PLoS One. 2014 Jan 2;9(1):e84694. doi: 10.1371/journal.pone.0084694 (PMC3879330; doi:10.1371/journal.pone.0084694)

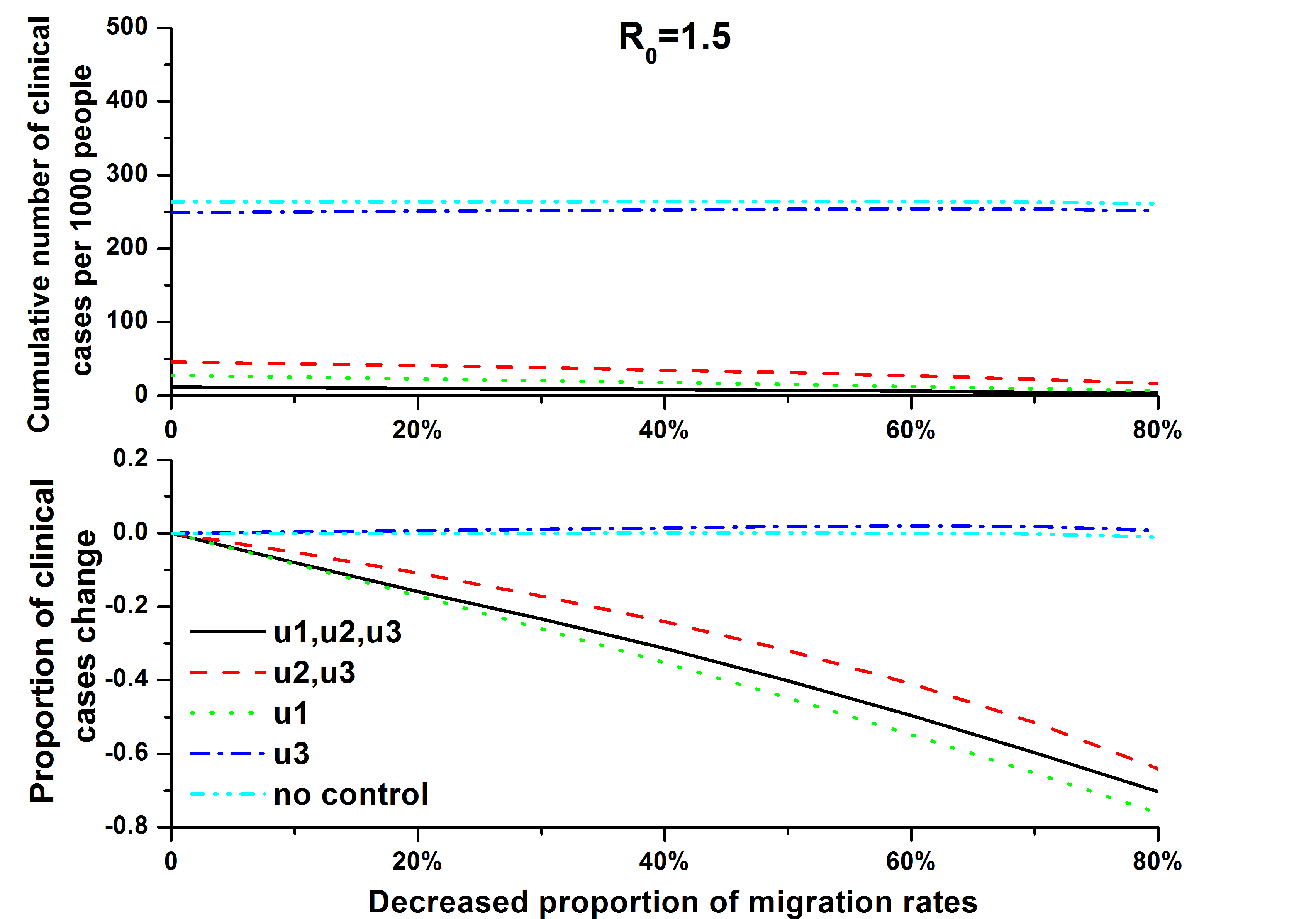

Supplement: Figure S1 — Cumulative number of and change in the proportion of clinical cases in region B as a function of migration rates between regions A and B, when = 1.5. Simulation parameters except are shown in Table 1. (TIF) [file pone.0084694.s001.tif]

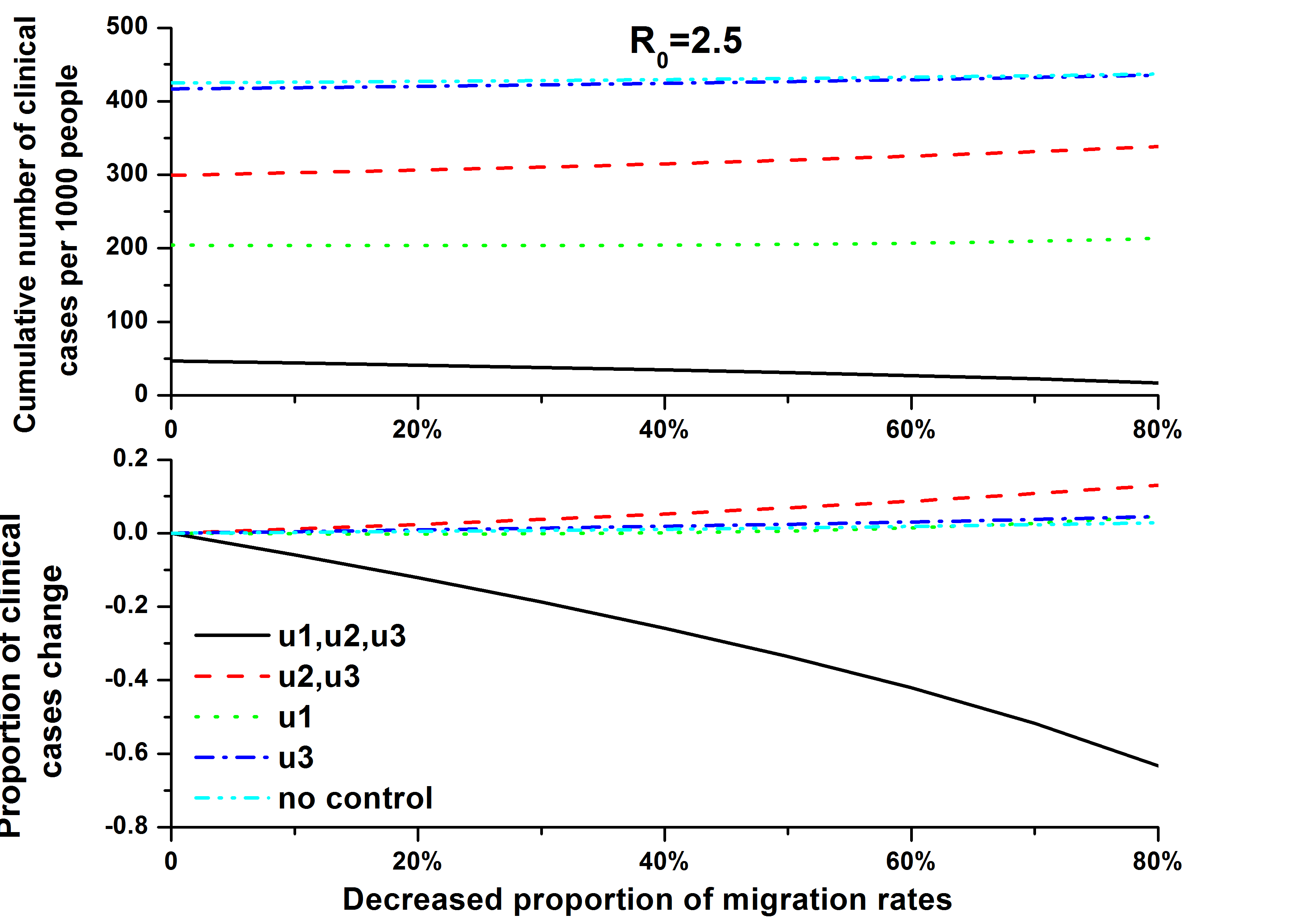

Supplement: Figure S2 — Cumulative number of and change in the proportion of clinical cases in region B as a function of migration rates between regions A and B, when = 2.5. Simulation parameters except are shown in Table 1. (TIF) [file pone.0084694.s002.tif]

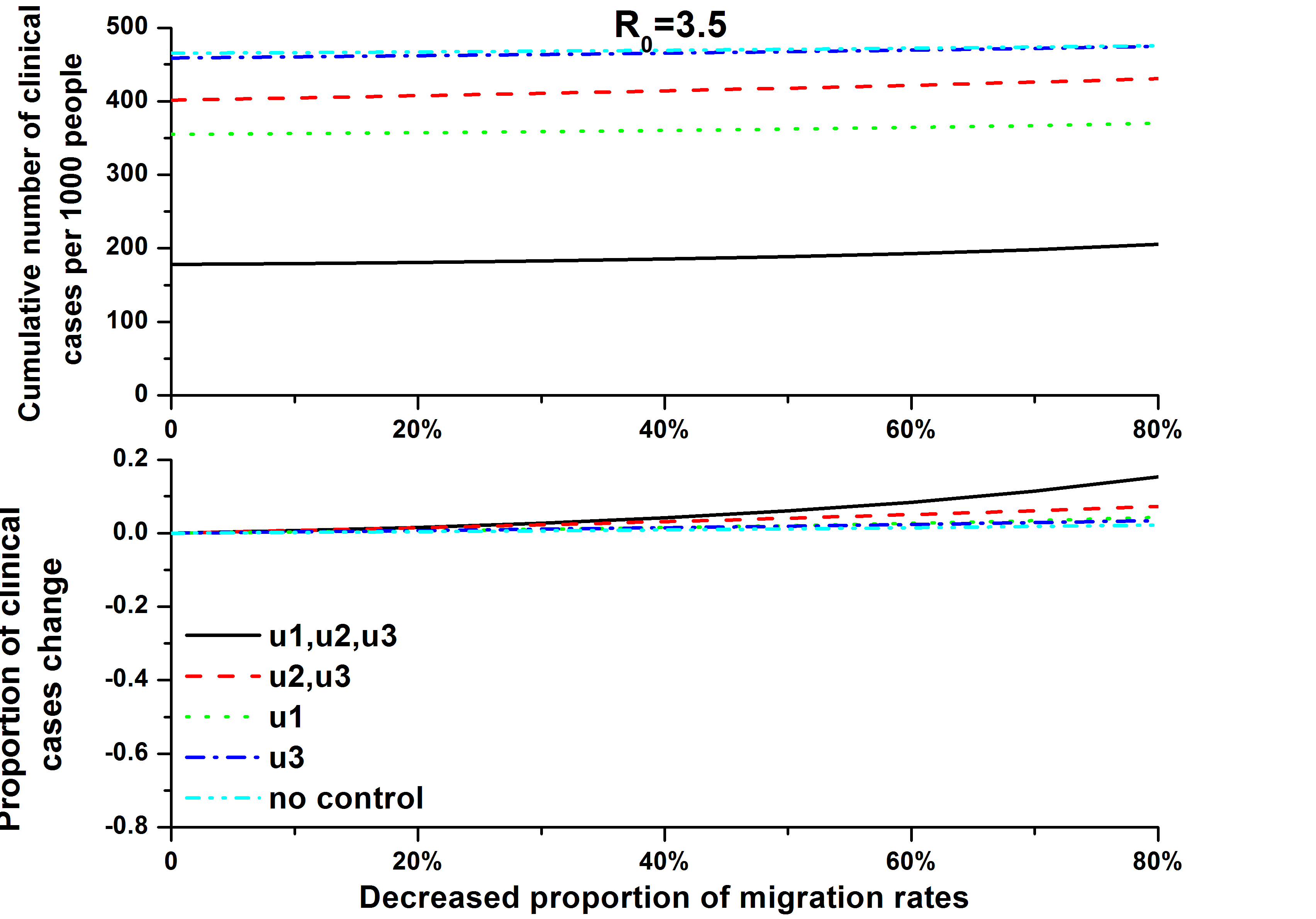

Supplement: Figure S3 — Cumulative number of and change in the proportion of clinical cases in region B as a function of migration rates between regions A and B, when = 3.5. Simulation parameters except are shown in Table 1. (TIF) [file pone.0084694.s003.tif]
