# Supplementary material for: Optimized Strategy for the Control and Prevention of Newly Emerging Influenza Revealed by the Spread Dynamics Model
Source: PLoS One. 2014 Jan 2;9(1):e84694. doi: 10.1371/journal.pone.0084694 (PMC3879330; doi:10.1371/journal.pone.0084694)
Supplement: Text S1 — The calculation method of Pontryagin’s Maximum Principle with unlimited antiviral resources. (PDF) [file pone.0084694.s004.pdf]

Supporting information text S1

The objective function (Eq. (3)) is converted into a problem of minimizing pointwise a Hamiltonian H by Pontryagin's Maximum Principle:

$$\begin{aligned} H &= I + \frac{c_1}{2}u_1^2 + \frac{c_2}{2}u_2^2 + \frac{c_3}{2}u_3^2 + \lambda_1\{-\beta S(1-\varepsilon_2 u_2)I + qA + \mu(NP_s - S)\} \\ &+ \lambda_2\{\beta S(t)((1-\varepsilon_2 u_2(t))I(t) + qA(t)) - kE(t) + \mu(N(t)P_E(t) - E(t))\} \\ &+ \lambda_3\{k(1-\rho)E(t) - \gamma_1 A(t) + \mu(N(t)P_A(t) - A(t))\} \\ &+ \lambda_4\{k\rho E(t) - \gamma_2 I(t) - \varepsilon_1 u_1(t)I(t) + \mu(N(t)P_I(t)(1-\varepsilon_3 u_3(t)) - I(t))\} \end{aligned} \tag{5}$$

According to this principle, if an optimal solution exists, then two conditions must be satisfied. First, continuous adjoint functions $\lambda_i(t)$ must exist that satisfy

$$\begin{cases} \dot{\lambda}_1(t) = -\dfrac{\partial H}{\partial S} = (\lambda_1 - \lambda_2)\beta((1-\varepsilon_2 u_2(t))I + qA) + \lambda_1\mu \\ \dot{\lambda}_2(t) = -\dfrac{\partial H}{\partial E} = \lambda_2(k+\mu) - \lambda_3 k(1-\rho) - \lambda_4 k\rho \\ \dot{\lambda}_3(t) = -\dfrac{\partial H}{\partial A} = (\lambda_1 - \lambda_2)\beta S q + \lambda_3(\gamma_1 + \mu) \\ \dot{\lambda}_4(t) = -\dfrac{\partial H}{\partial I} = \beta S(\lambda_1 - \lambda_2)(1-\varepsilon_2 u_2(t)) + \lambda_4(\gamma_2 + \varepsilon_1 u_1(t) + \mu) - 1 \end{cases} \tag{6}$$

with transversality conditions $\lambda_i(t_f) = 0, \quad i = 1, \ldots, 4$. Second, the Hamiltonian H must be minimized with respect to the optimal control, satisfying the following optimality conditions:

$$\begin{cases} \dfrac{\partial H}{\partial u_1} = c_1 u_1 - \lambda_4 \varepsilon_1 I = 0 \\ \dfrac{\partial H}{\partial u_2} = c_2 u_2 + (\lambda_1 - \lambda_2)\beta S \varepsilon_2 I = 0 \\ \dfrac{\partial H}{\partial u_3} = c_3 u_3 - \lambda_4 \mu N P_I \varepsilon_3 = 0 \end{cases} \tag{7}$$

Solving Eq. (7) for u_1, u_2, and u_3, we obtain:

$$u_1 = \frac{\lambda_4 \varepsilon_1 I}{c_1}, u_2 = \frac{(\lambda_2 - \lambda_1)\beta S \varepsilon_2 I}{c_2}, u_3 = \frac{\lambda_4 \mu N P_I \varepsilon_3}{c_3} \quad (8)$$

When this equation is combined with the restrictions of the control parameters, $0 < u_i(t) < 1, i = 1, 2, 3, t \in (t_0, t_f)$ (adjusted according to the specific assumptions), the control parameters of the optimal solution are:

$$\begin{cases} u_1^* = \min\{\max(0, \frac{\lambda_4 \varepsilon_1 I}{c_1}), 1\} \\ u_2^* = \min\{\max(0, \frac{(\lambda_2 - \lambda_1)\beta S \varepsilon_2 I}{c_2}), 1\} \\ u_3^* = \min\{\max(0, \frac{\lambda_4 \mu N P_I \varepsilon_3}{c_3}), 1\} \end{cases} \quad (9)$$

The integrand in Eq. (3) is a convex function of (u_1, u_2, u_3), and the state equation in Eq. (1) satisfies the Lipshitz property. The existence and uniqueness of the optimal controls can be proven according to the literature [1,2].

Given the initial value of the state equation according to the actual situation, the problem of solving the optimal solution of the system converts into a two-point boundary value problem. Because Eqs. (3) and (6) are coupled nonlinear differential equations, it is difficult to find analytic solutions. Using a fourth-order Runge-Kutta method, we created an iterative program to simulate numerical solutions for $u_1^*(t)$, $u_2^*(t)$, and $u_3^*(t)$ that satisfied the restrictions. Finally, the antiviral resource consumption can be calculated according to:

$$M = \int_{t_0}^{t_f} u_1(t) I(t) dt \quad (10)$$

References

1. Fister KR, Lenhart S, McNally JS (1998) Optimizing chemotherapy in an HIV model.

Electroic Journal of Differential Equations 32: 1-12.

2. Lenhart S and Workman JT (2007) Optimal control applied to biological models. Charlottesville: Chapman & Hall/CRC. 261p.
