# Supplementary material for: Optimized Strategy for the Control and Prevention of Newly Emerging Influenza Revealed by the Spread Dynamics Model
Source: PLoS One. 2014 Jan 2;9(1):e84694. doi: 10.1371/journal.pone.0084694 (PMC3879330; doi:10.1371/journal.pone.0084694)
Supplement: Text S2 — The calculation method of Pontryagin’s Maximum Principle with limited antiviral resources. (PDF) [file pone.0084694.s005.pdf]

Supporting information text S2

For exploring the optimal control strategies under the condition of limited antiviral stockpiles, Eq. (1) was changed as shown in Eq. (11), to increase the variable of the antiviral resource consumption $W(t)$. The antiviral resources are limited by $W(t)$ during the simulation time, and the initial and terminal values are 0 and W_T, respectively.

$$
\begin{cases}
\dot{S}(t) = -\beta S(t)((1-\varepsilon_2 u_2(t))I(t) + qA(t)) + \mu(N(t)P_S(t) - S(t)) \\
\dot{E}(t) = \beta S(t)((1-\varepsilon_2 u_2(t))I(t) + qA(t)) - kE(t) + \mu(N(t)P_E(t) - E(t)) \\
\dot{A}(t) = k(1-\rho)E(t) - \gamma_1 A(t) + \mu(N(t)P_A(t) - A(t)) \\
\dot{I}(t) = k\rho E(t) - \gamma_2 I(t) - \varepsilon_1 u_1(t)I(t) + \mu(N(t)P_I(t)(1-\varepsilon_3 u_3(t)) - I(t)) \\
\dot{R}(t) = \gamma_1 A(t) + \gamma_2(1-d)I(t) + \varepsilon_1 u_1(t)I(t) + \mu(N(t)P_R(t) - R(t)) \\
\dot{D}(t) = \gamma_2 dI(t) \\
\dot{N}(t) = -\gamma_2 dI(t) \\
\dot{W}(t) = I(t)u_1(t)
\end{cases} \tag{11}
$$

The optimization objective function is unchanged and, based on Eqs. (11) and (4), the Hamiltonian is defined as:

$$
\begin{aligned}
H = {} & I + \frac{c_1}{2}u_1^2 + \frac{c_2}{2}u_2^2 + \frac{c_3}{2}u_3^2 + \lambda_1\{-\beta S(1-\varepsilon_2 u_2)I + qA + \mu(NP_s - S)\} \\
& + \lambda_2\{\beta S(t)((1-\varepsilon_2 u_2(t))I(t) + qA(t)) - kE(t) + \mu(N(t)P_E(t) - E(t))\} \\
& + \lambda_3\{k(1-\rho)E(t) - \gamma_1 A(t) + \mu(N(t)P_A(t) - A(t))\} \\
& + \lambda_4\{k\rho E(t) - \gamma_2 I(t) - \varepsilon_1 u_1(t)I(t) + \mu(N(t)P_I(t)(1-\varepsilon_3 u_3(t)) - I(t))\} \\
& + \lambda_5\{I(t)u_1(t)\}
\end{aligned} \tag{12}
$$

As in Text S1, the optimal solution of the dynamic control variables can be deduced as

$$
u_1^* = \min\{\max(0, \frac{\lambda_4\varepsilon_1 I - \lambda_5 I}{c_1}), 1\}
$$

$$u_2^* = \min\{\max(0, \frac{(\lambda_2 - \lambda_1)\beta S \varepsilon_2 I}{c_2}), 1\} \quad (13)$$

$$u_3^* = \min\{\max(0, \frac{\lambda_4 \mu N P_I \varepsilon_3}{c_3}), 1\}$$

$\lambda_5(t)$ is an unknown constant that directly determines the size of W_T. Therefore, the problem converts into searching for a suitable value of $\lambda_5(t)$ and the optimal control process corresponding to $\lambda_5(t)$. The optimal control process is solved by a variable step-size search method and by solving for the optimal solution (Text S1).
