# Supplementary material for: Optimized Strategy for the Control and Prevention of Newly Emerging Influenza Revealed by the Spread Dynamics Model
Source: PLoS One. 2014 Jan 2;9(1):e84694. doi: 10.1371/journal.pone.0084694 (PMC3879330; doi:10.1371/journal.pone.0084694)
Supplement: Text S3 — Basic assumptions of model. (PDF) [file pone.0084694.s006.pdf]

Supporting information text S3

Intensities of the control measures

Because it was difficult to detect an individual in class E or A, we assumed that epidemic intervention could only be used for clinical patients. $u_1(t)$ represented the proportion of clinical patients who accepted antiviral treatment ($u_1(t) \in (0,1)$), with ε_1 as the efficacy of this treatment ($\varepsilon_1 \in (0,1)$). $u_2(t)$ represented the proportion of isolated clinical patients, with ε_2 as the efficacy of this isolation ($\varepsilon_2 \in (0,1)$). $u_3(t)$ represented the proportion of flowing clinical patients who underwent immigration detection ($u_3(t) \in (0,1)$), with ε_3 as the efficacy of immigration detection ($\varepsilon_3 \in (0,1)$).

Model assumptions

We made the following assumptions about model:

1. The total population of region B was 100,000. All of them were susceptible.
2. The influenza epidemic began in region A, and then spread to region B.
3. The upper limits of the intensities of antiviral treatment, immigration detection, and isolation were 0.9, 0.9, and 0.6, respectively. The limit for isolation was lower than the other limits because it was hard to execute effectively. Thus, $0 < u_1(t) < 0.9, 0 < u_2(t) < 0.6, 0 < u_3(t) < 0.9$.
4. According to medical research, both clinical and subclinical infections are possible from the same influenza virus source [1]. There is no research to indicate the specific proportion of clinical infection of influenza A (H1N1).

We speculate that the number of clinical cases is approximately equal to the number of subclinical cases from literature [2,3]. Therefore, we assumed that $\rho = 0.5$.

5. The infectiousness and infectious period of subclinical individuals have not been statistically validated with medical observations. Because these patients lack coughing, sneezing, and other obvious influenza symptoms, their infectiousness will be much weaker than that of clinical individuals. We assumed that the infectious period for a subclinical individual was 5 days, and we used infectious parameters from the 1918 influenza outbreak [4].
6. Three weight values were defined to balance the cost of control measures and loss of influenza: $c_1 = 5$, $c_2 = 20$, and $c_3 = 20$.
7. The simulation time was set to 200 days. The infectiousness levels of various new influenza strains may differ considerably [3,5]. We assumed an R_0 for new influenza of 3.0. The transmission rate β can be solved from Eq. (2).

The population mobility and the weights and effectiveness levels of the different control measures were difficult to determine and were subjected to sensitivity analyses.

References

1. Yang J, Yang F, Huang F, Wang JW, Jin Q (2009) Subclinical infection with the novel influenza A(H1N1) virus. Clin Infect Dis 49:1622-1623.

2. Girard MP, Tam JS, Assossou OM, Kieny MP (2010) The 2009 A (H1N1) influenza virus pandemic: A review. Vaccine 28: 4895-4902.
3. Fraser C, Donnelly CA, Cauchemez S, Hanage WP, Van Kerkhove MD, et al. (2009) Pandemic potential of a strain of influenza A (H1N1): early findings. Science 324: 1557-1561.
4. Chowell G, Ammon CE, Hengartner NW, Hyman JM (2006) Transmission dynamics of the great influenza pandemic of 1918 in Geneva, Switzerland: Assessing the effects of hypothetical interventions. J Theor Biol 241: 193-204.
5. Poletti P, Ajelli M, Merler S, et al. (2011) The Effect of Risk Perception on the 2009 H1N1 Pandemic Influenza Dynamics. PLOS ONE 6: e16460.
