# Supplementary material for: Optimized Strategy for the Control and Prevention of Newly Emerging Influenza Revealed by the Spread Dynamics Model
Source: PLoS One. 2014 Jan 2;9(1):e84694. doi: 10.1371/journal.pone.0084694 (PMC3879330; doi:10.1371/journal.pone.0084694)
Supplement: Text S4 — Sensitivity analysis of migration rates. (DOC) [file pone.0084694.s007.doc]

**Supporting information text S4**

**Sensitivity analysis of migration rates**

Recently, studies have shown that migration rates between epidemic and nonepidemic regions decrease spontaneously when influenza breaks out. The proportion of decline varies between different regions, from 49.9% in the UK [1], to 59% in the United States [2] and 63.8% in Hong Kong [3] (figures from 2009). Therefore, we investigated the role of migration rates in an influenza epidemic period with different *R*_0_ values via a sensitivity analysis. Because the decrease in migration rates does not differ significantly during different stages of an epidemic [3], we assumed that the change in migration rates would be constant during the influenza epidemic period. We examined the change in the number of clinical cases under ideal control strategies when the migration rates between region A and region B were in the range of 0 to 0.8 with different values of *R*_0_ (see Fig. S1, Fig. S2, Fig. S3).

The number of clinical cases did not significantly change with different migration rates. When the control strategies were relatively effective (cumulative clinical cases per thousand people < 100), the number of clinical cases was positively correlated with migration rates, and the proportion of change was significant. The major reason for this result is that region A has a much higher proportion of clinical cases than region B, and immigration detection cannot effectively prevent clinical cases from entering region B. When the control strategies are not effective (cumulative clinical cases per thousand people > 100), the number of clinical cases was negatively correlated with migration rates, and the proportion of change was not significant. The major reason for this result is that regions A and B have similar proportions of clinical cases, and the epidemic ends early in region A. Some infectious people entering region A cannot return to region B due to immigration detection; thus, the larger the migration rates are, the fewer clinical cases there will be in region B.

**References**

1. Rubin GJ, Amlot R, Page L, Wessely S (2009) Public perceptions, anxiety, and behaviour change in relation to the swine flu outbreak: cross sectional telephone survey. Brit Med J 339: b2651

2. Jones JH, Salathe M (2009) Early assessment of anxiety and behavioral response to novel swine-origin influenza A(H1N1). PLOS ONE 4: e8032.

3. Lau JT, Griffiths S, Au DW, Choi KC (2011) Changes in knowledge, perceptions, preventive behaviours and psychological responses in the precommunity outbreak phase of the H1N1 epidemic. Epidemiol Infect 139: 80-90.
